# The Interaction Between N^6^-Methyladenosine Modification and Non-Coding RNAs in Gastrointestinal Tract Cancers

**DOI:** 10.3389/fonc.2021.784127

**Published:** 2022-01-07

**Authors:** Lin Yao, Chang-Feng Man, Rong He, Lian He, Jia-Bin Huang, Shou-Yan Xiang, Zhe Dai, Xiao-Yan Wang, Yu Fan

**Affiliations:** ^1^ Cancer Institute, The Affiliated People’s Hospital of Jiangsu University, Zhenjiang, China; ^2^ Digestive Department, The Affiliated Suqian first People’s Hospital of Nanjing Medical University, Suqian, China

**Keywords:** m^6^A modification, non-coding RNAs (ncRNAs), gastrointestinal tract cancers, colorectal cancer, long non-coding RNA (lncRNA)

## Abstract

N^6^-methyladenosine (m^6^A) is the most common epigenetic modification of eukaryotic RNA, which can participate in the growth and development of the body and a variety of physiological and disease processes by affecting the splicing, processing, localization, transport, translation, and degradation of RNA. Increasing evidence shows that non-coding RNAs, particularly microRNA, long non-coding RNA, and circular RNA, can also regulate the RNA m^6^A modification process by affecting the expression of m^6^A-related enzymes. The interaction between m^6^A modification and non-coding RNAs provides a new perspective for the exploration of the potential mechanism of tumor genesis and development. In this review, we summarize the potential mechanisms and effects of m^6^A and non-coding RNAs in gastrointestinal tract cancers.

## Background

Tumors of the digestive system are the most common malignant tumors, mainly including colorectal cancer, gastric cancer, liver cancer, pancreatic cancer, esophageal cancer, and gallbladder cancer. Since the early symptoms of gastrointestinal tract cancers are not obvious, it is often easier to be ignored. Patients are often treated in a late-stage, which leads to difficult treatment and poor prognosis. An in-depth study of the molecular mechanism of the occurrence and development of gastrointestinal tract cancers is helpful to find targets for early diagnosis and treatment, so as to improve the level of comprehensive diagnosis and treatment of gastrointestinal tract cancers.

**Figure 1:**
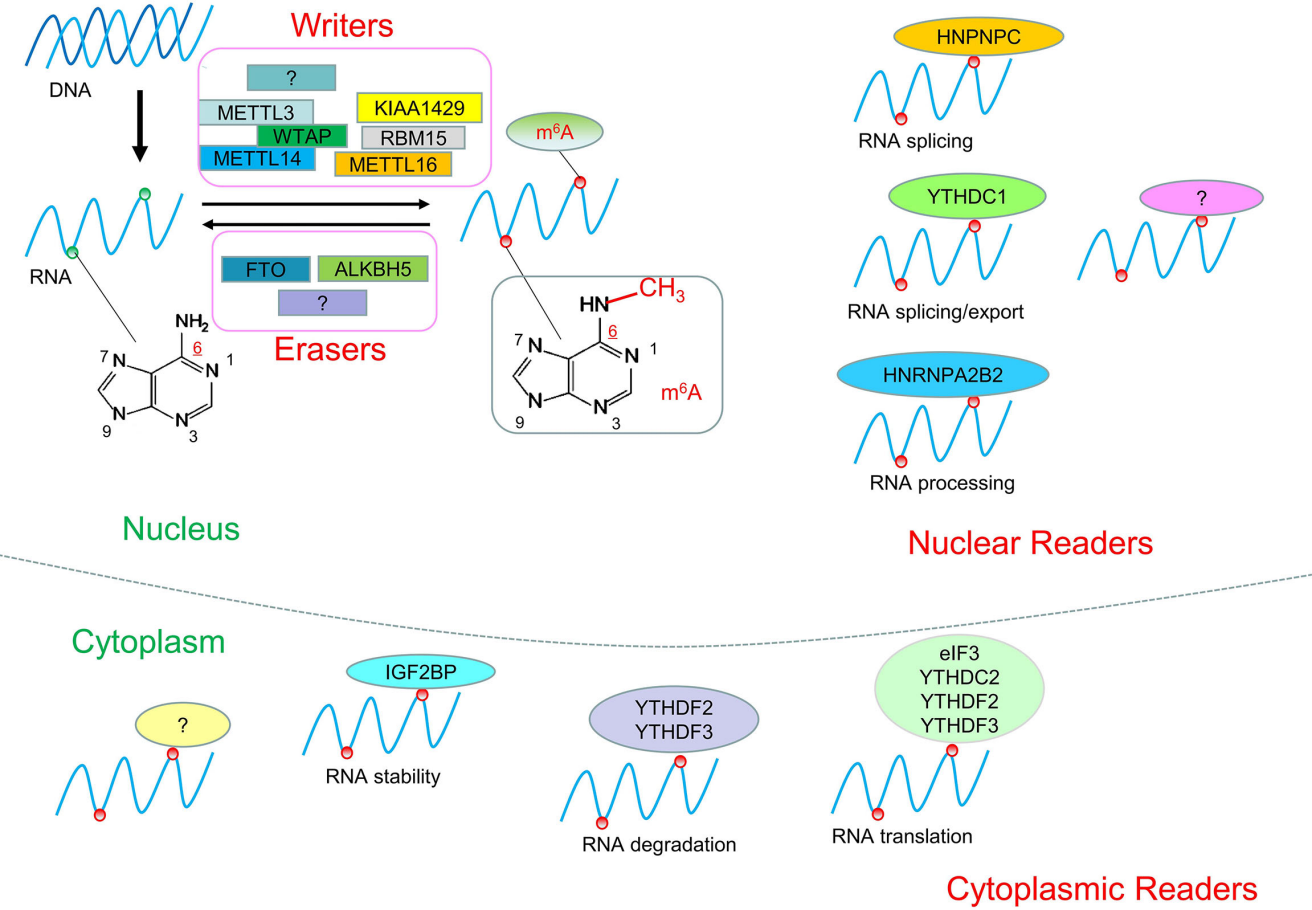
RNA m^6^A modification. RNA m^6^A modification is a dynamic and reversible process. RNA could be methylated by Writers and demethylated by Erasers. RNA modified by m^6^A can be recognized by Readers to regulate RNA processing, localization, stability, translation, and degradation.

Epigenetics is a branch of genetics that studies the heritable changes in gene expression without changes in the nucleotide sequence of genes. It mainly includes DNA/RNA methylation, histone modification, chromatin remodeling, and non-coding RNAs regulation. Known eukaryotic RNA has more than 100 kinds of modifications, among which the common ones include: N^1^-methyladenosine (m^1^A), m^6^A, and 5-methylcytosine (m^5^C), of which RNA m^6^A is the most common eukaryotic RNA Epigenetic modification, which is involved in many key processes of mammalian growth and development and disease (1–4). N^6^-methyladenosine (m^6^A) refers to the methylation of the nitrogen atom at the sixth position of the RNA molecule adenine. It is known that m^6^A modification is the most common in mRNAs, and can also appear in tRNAs, rRNAs, and non-coding RNAs. It regulates gene expression by influencing various metabolic pathways such as RNA processing, transport, translation, and degradation, thus participating in various physiological and pathological processes (1, 5). It is known that m^6^A is involved in the growth and development of the body (6), learning and memory (7), immune response (8), and disease occurrence (9). The level of RNA methylation in the body is regulated by three enzymes (Writers, Erasers, Readers) in a dynamic equilibrium state (**Figure 1**). When the balance is broken, it will cause disease. Recently, the study of epigenetic modification, especially RNA m^6^A, in prognosis prediction and treatment of tumors has been increasing. In lung and bladder cancer, for example, high expression of the m^6^A-related enzyme METTL3 indicates a worse prognosis (10, 11). Meclofenamic acid (MA) and its ethyl ester derivative MA2 can selectively inhibit the demethylation of m^6^A-related enzyme FTO and have been used in the treatment of glioma (12, 13).

In recent years, research on RNA m^6^A modification has become more and more popular, but the main focus is on m^6^A modification of mRNAs, and less attention has been paid to m^6^A modification of non-coding RNAs. Recently, a growing body of evidence suggests that RNA m^6^A modification and non-coding RNAs play an important role in the occurrence and development of tumors, which opens a new door for the diagnosis and treatment of malignant tumors. In this review, we mainly elaborated on the role of m^6^A and non-coding RNAs interaction in gastrointestinal tract cancers.

## Research Progress of RNA m^6^A Modification

Interest in RNA m^6^A modification has been ongoing since its discovery in the 1970s (14, 15). The discovery of FTO in 2011 revealed that m^6^A modification is a dynamic and reversible process (16). In 2012, the discovery of m^6^A modification detection methods m^6^A-seq and MeRIP-seq brought about a great turning point in the study of RNA m^6^A modification (17, 18). It has been found that m^6^A modification sites are often located in the consensus sequence RRACH (R = G or A and H = A, C, or U), which tends to be found in 3′untranslated regions (3′UTRs) and stop codons (17, 18). The level of m^6^A modification in the human body is in a dynamic equilibrium state, and its abnormally high or low level will lead to the occurrence of disease. For example, the level of m^6^A modification is increased in hepatocellular carcinoma and decreased in cervical carcinoma (19, 20). An increasing amount of evidence suggests that abnormal m^6^A modifications are associated with many types of cancer, including lung, breast, cervical, bladder, glioma, and others (10, 21–24). The dynamic reversible process of m^6^A was mainly related to the regulation of three types of enzymes: Writers, Erasers, and Readers (**Table 1** and **Figure 1**).

**Table 1:** Related enzymes and functions of RNA m^6^A.

Type	Enzyme	Function	References
Writers	METTL3	catalytic core, catalyzes m^6^A modification of RNA *in vivo* and *in vitro*	(25)
	METTL14	RNA binding platform that specifically recognizes binding target RNA: Stabilize the METTL3 structure	(25)
	WTAP	promotes recognition of m^6^A modification sites and activates the methyltransferase complex	(26)
	KIAA1429	aggregates the methyltransferase complex at a specific site	(27)
	ZC3H13	promotes localization of the m^6^A complex into the nucleus	(28)
	METTL16	catalyzes m^6^A methylation of RNA	(29)
	RBM15/15B	helps to gather the enzyme complex to the target site	(30)
	ZCCHC4	is involved in methylation of human 28S rRNA.	(31)
Erasers	FTO	RNA demethylase	(32)
	ALKBH5	RNA demethylase	(32)
Readers	YTHDC1	promotes the transport and splicing of RNA	(33)
	YTHDC2	promotes RNA translation	(34)
	YTHDF1	promotes RNA translation	(35)
	YTHDF2	promotes RNA degradation	(36)
	YTHDF3	interaction with YTHDF1 enhances its ability to promote mRNA translation or interaction with YTHDF2 enhances its ability to promote mRNA degradation	(37)
	HNRNPC	regulates the selective splicing and structural changes of RNA	(38)
	HNRNPA2B1	regulates the selective splicing and structural changes of RNA	(38)
	eIF3	promotes RNA translation	(35)
	IGF2BP1/2/3	increases RNA stability and translation	(39)

Writers are methyltransferases, which use S-adenosylmethionine (SAM) as a methyl donor to mediate the process of methylation modification of RNA (**Table 1**). Writers mainly exist in the form of complexes to play their catalytic role (40). The currently known methyltransferase complex components mainly include METTL3 (methyltransferase-like protein 3), METTL14 (methyltransferase-like protein 14), WTAP (Wilms tumor suppressor-1-associated protein). Among them, METTL3 is the catalytic core, and METTL14 is the RNA binding platform (25); WTAP itself does not have methylation activity. It co-localizes in nuclear speckles with the METTL3-METTL14 heterodimer in the nucleus, which helps the methylase complex quickly recognize the modification site of m^6^A and activate the METTL3-METTL14 complex (26). Writers also include KIAA1429, ZC3H13 (Zinc finger CCCH-type containing 13), RBM15/15B(RNA binding motif protein 15/15B), ZCCHC4 (zinc finger CCHC-type containing 4), and METTL16 (27, 29, 30). KIAA1429(also known as VIRMA, Vir like m^6^A methyltransferase associated) recruits the METTL3-METTL14-WTAP complex to specific sites (41). ZC3H13 promotes the localization of the m^6^A complex into the nucleus (28). ZC3H13 plays an important role in the progression of colorectal, breast, and kidney cancers (42–44). RBM15/15B contributes to the aggregation of the transferase complex to the target (30), which is involved in the progression of hematopoietic diseases, HCC, and laryngeal cancer (45–47). In addition, a new methyltransferase, ZCCHC4, has been discovered in recent years. ZCCHC4 is mainly involved in the methylation of human 28S rRNA and is highly expressed in HCC (31).

Erasers are demethylases that mediate the process of demethylation modification of RNA (**Table 1**). Erasers mainly include FTO (fat mass and obesity associated protein) and ALKBH5 (ALKB homolog 5). Both belong to the AlkB family and rely on ferric divalent ions and α-ketoglutaric acid for their demethylation function (32). Studies have shown that FTO is related to the occurrence and development of multiple malignant tumors such as breast cancer (48), glioblastoma (49), and acute myeloid leukemia (50).

Readers are m^6^A binding proteins, which can specifically recognize RNA m^6^A modification site information to perform different biological functions (**Table 1**). Readers mainly include YTHDF1-3 and YTHDC1-2 containing the YTH domain, HNRNPC, and HNRNPA2B1 from the heterogeneous nuclear ribonucleoprotein (HNRNP) family, as well as eIF3 (eukaryotic translation initiation factor), IGF2BP1/2/3 (insulin-like growth factor 2 mRNA-binding protein 1/2/3) (39). The YTH domain at the carboxyl terminal of YTHDF1-3 protein can bind to RNA, and the P/Q/N rich region at the amino terminal of YTHDF1-3 protein can bind to the m^6^A site of mRNA (35, 51). YTHDC1 promotes the transport and splicing of m^6^A-modified RNA (33); YTHDC2 can promote RNA translation (34); YTHDF1 interacts with translation initiation factor eIF3 to stimulate translation of the corresponding RNA (35); YTHDF2 promotes RNA degradation (36); YTHDF3 has a bidirectional regulation effect. When it binds to YTHDF1, it can enhance the ability of YTHDF1 to promote RNA translation, and when it binds to YTHDF2, it can also enhance the ability of YTHDF2 to promote RNA degradation (37); HNRNPC and HNRNPA2B1 regulate the selective splicing and structural changes of RNA (38).

## Non-Coding RNAs

Non-coding RNAs (ncRNAs) are non-protein-coding RNAs, which mainly include micro RNAs (miRNAs), circular RNAs (circRNAs), and long noncoding RNAs (lncRNAs). Non-coding RNA can participate in the protein expression process in a variety of ways.

MiRNAs are endogenous non-coding RNA molecules of approximately 20-22 nucleotides in length. Firstly, the miRNA gene is transcribed into the precursor miRNA (pre-miRNA). Subsequently, in the cytoplasm, the pre-miRNA is cleaved by the Dicer enzyme into mature miRNA (52). Functionally, miRNAs bind to the 3 ‘untranslated region (UTR) of the target gene, resulting in mRNA degradation of the target gene or translation inhibition at the post-transcriptional level of the target gene (53);

CircRNAs are formed by reverse splicing of precursor mRNA(pre-mRNA) (54). There are three main cyclization mechanisms of circRNAs: Intron reverse complementary sequence driven cyclization; RNA binding proteins drive cyclization; Lasso drive cyclization (55–57). Functionally, circRNAs mainly achieve their epigenetic regulation through the following pathways: circRNAs as ceRNA sponges miRNAs to block or reduce the inhibition of miRNAs on target genes; circRNAs can regulate transcription and splicing of target genes. In addition, circRNAs can also be directly involved in protein-protein interactions. (57–60);

LncRNAs are non-coding RNAs larger than 200 nucleotides in length. The synthesis process of lncRNAs is similar to mRNA, with 5 ‘-terminated 7-methylguanosine cap and 3’ -terminated polyadenylate tail. But unlike the mRNA that encodes the protein, lncRNAs don’t have an open reading frame (ORF) (61, 62). LncRNAs can interact with different molecules to exert epigenetic regulation: LncRNAs can regulate gene transcription by reshaping chromatin or directly contacting RNA polymerase and transcription factors; LncRNAs can bind to mRNA and affect its processing and translation; LncRNAs bind to proteins to regulate protein activity; Recent studies have found that lncRNAs can also serve as ceRNA as miRNA sponges. (63–67).

## m^6^A and ncRNAs

In recent years, many studies have proved that not only m^6^A modification exists in mRNAs, but many ncRNAs are also regulated by m^6^A modification (68–70). The m^6^A modification of ncRNAs can not only regulate its processing, splicing, and expression but also affect its positioning and stability. For example, in pancreatic cancer, IGF2BP2 as Reader recognizes m^6^A-modified lncRNA DANCR, which promotes the progression of pancreatic cancer by improving the stability of DANCR (71). Similarly, ncRNAs can also regulate the process of RNA m^6^A modification by affecting the expression of m^6^A-related enzymes. For example, in hepatoblastoma (HB), microRNA miR-186 negatively regulates the expression of METTL3, thereby inhibiting the proliferation and invasion of HB cells (72).

## The role of m^6^A Interaction With ncRNAs in Gastrointestinal Tract Cancers

Currently, an increasing number of studies have proved that m^6^A and ncRNAs are associated with the occurrence and development of a variety of tumors. RNA m^6^A modification and ncRNAs can affect the expression of downstream oncogenes or tumor suppressor genes through a variety of pathways, thus influencing tumor progression (**Table 2**).

**Table 2:** Interaction between m^6^A and ncRNAs in gastrointestinal tract cancers.

Cancer	Enzyme	Type	Enzyme expression	ncRNA	ncRNA expression	Mechanism	Biological function	References
Colorectal cancer	METTL3	Writer	Up	A subset of miRNAs (miR-483, miR-676, and miR-877)	Up	METTL3 promotes the post-transcriptional processing of subsets of miRNAs	promotes tumor growth	(73)
	METTL3	Writer	Up	lncRNA RP11	Up	METTL3 promotes the expression of lncRNA RP11	promotes CRC cells migration, invasion, and EMT	(70)
	METTL3	Writer	Up	microRNA miR-1246	Up	METTL3 promotes miR-1246 maturation	promotes the migration and invasion of CRC cells	(69)
	METTL3	Writer	Up	lncRNA LINC01605	Up	LINC01605 promotes m^6^A modification of SPTBN2 mRNA by METTL3	promotes the proliferation, migration and invasion of CRC cells	(74)
	METTL3	Writer	Up	lncRNA LBX2-AS1	Up	METTL3 promotes lncRNA LBX2-AS1 expression	promotes the proliferation, migration, invasion and 5-FU resistance of CRC cells	(75)
	METTL14	Writer	Down	lncRNA XIST	Up	METTL14 promotes the degradation of lncRNA XIST	inhibits proliferation and invasion of CRC cells	(76)
	METTL14	Writer	Down	microRNA miR-375	Down	METTL14 promotes miR-375 processing	inhibits the migration and invasion of CRC cells	(77)
	ALKBH5	Eraser	Up	lncRNA NEAT1	Up	ALKBH5 promotes the expression of lncRNA NEAT1	promotes the proliferation and migration of colon cancer cells	(78)
	YTHDC1	Reader	–	circNSUN2	Up	YTHDC1 promotes the export of circNSUN2 from the nucleus to the cytoplasm	promotes liver metastasis of CRC	(68)
	YTHDF3	Reader	Up	lncRNA GAS5	Down	lncRNA GAS5 inhibits the transcriptional expression of YTHDF3	promotes the proliferation and invasion of CRC cells	(79)
	IGF2BP2	Reader	Up	lncRNA LINRIS	Up	LINRIS blocks IGF2BP2 degradation	promotes MYC-mediated glycolysis and CRC cells proliferation	(80)
	IGF2BP2	Reader	–	lncRNA LINC00460	Up	The formation of LINC00460/DHX9/IGF2BP2 complex enhances the stability of HMGA1 mRNA	promotes the proliferation, migration and invasion of CRC cells	(81)
Gastric cancer	METTL3	Writer	–	lncRNA LINC00470	Up	LINC00470 promotes the interaction between METTL3 and PTEN mRNA, thereby promoting the degradation of PTEN mRNA and decreasing its expression	promotes the proliferation, migration and invasion of gastric cancer cells	(82)
	METTL3	Writer	–	lncRNA ARHGAP5-AS1	Up	lncRNA ARHGAP5-AS1 promotes ARHGAP5 transcription and improves the stability of ARHGAP5 mRNA by recruiting METTL3 to modify ARHGAP5 mRNA with m^6^A	promotes chemotherapy resistance in gastric cancer	(83)
	METTL3	Writer	Up	miR-4429	Down	miR-4429 targets METTL3 and prevents its stabilization of oncogene SEC62 mRNA	inhibits GC proliferation and promote apoptosis	(84)
	METTL3	Writer	Up	lncRNA BLACAT2	Up	BLACAT2 promotes METTL3 expression	promotes the progression of gastric cancer	(85)
	KIAA1429	Writer	Up	lncRNA LINC00958	Up	KIAA1429 up-regulates the expression of LINC00958	promotes aerobic glycolysis of GC cells	(86)
	ALKBH5	Eraser	Up	lncRNA NEAT1	Up	ALKBH5 promotes the expression of lncRNA NEAT1	promotes the invasion and metastasis of gastric cancer	(87)
Liver cancer	METTL3	Writer	Up	lncRNA LINC00958	Up	METTL3 upregulates the expression of LINC00958 by stabilizing the RNA transcription of LINC00958	promotes proliferation, migration, invasion and lipogenesis of HCC cells	(88)
	METTL3	Writer	Up	circRNA-SORE	Up	METTL3 increases the stability of circRNA-SORE RNA	induces sorafenib resistance in HCC	(89)
	METTL3	Writer	Up	microRNA miR-186	Down	miR-186 negatively regulates METTL3 expression	inhibits the proliferation and invasion of HB cells	(72)
	METTL3	Writer	Up	circ-ARL3	Up	METTL3 promotes reverse splicing and formation of circ-ARL3	promotes the proliferation and invasion of HBV^+^ HCC cells	(90)
	METTL3	Writer	Up	lncRNA ILF3-AS1	Up	ILF3-AS1 increases the methylation level of ILF3 by recruiting METTL3, thereby inhibiting degradation of ILF3	promotes the proliferation, migration and invasion of HCC cells.	(91)
	METTL3	Writer	Up	lncRNA NIFK−AS1	Up	METTL3 promotes lncRNA NIFK AS1 expression	promotes the growth and invasion of HCC cells and the resistance to sorafenib	(92)
	METTL3	Writer	Up	LncRNA MEG3	Down	METTL3 inhibits MEG3 expression	promotes the growth and invasion of HCC cells	(93)
	KIAA1429	Writer	Up	lncRNA GATA3-AS	–	lncRNA GATA3-AS targeting promotes m^6^A modification of GATA3 pre-mRNA by KIAA1429, resulting in decreased stability of GATA3 pre-mRNA and decreases expression of GATA3	promotes the invasion and migration of HCC cells	(94)
	KIAA1429	Writer	Up	circDLC1	Down	KIAA1429 inhibits the expression of circDLC1	promotes the proliferation and movement of HCC cells	(95)
	WTAP	Writer	Up	miR-139-5p	Down	miR-139-5p inhibits the expression of WTAP	Inhibits the EMT process of HCC	(96)
	YTHDF1	Reader	Up	lncRNA TPTEP1	–	YTHDF1-mediated increases GNAS translation inhibited the interaction between lncRNA TPTEP1 and STAT3	promotes LPS-induced growth and invasion of HCC cells	(97)
Pancreatic cancer	METTL3	Writer	Up	lncRNA LINC00857	Up	METTL3 enhances the stability of LINC00857 RNA, resulting in up-regulation of its expression	promotes the proliferation of pancreatic cancer cells and inhibit apoptosis	(98, 99)
	METTL3	Writer	Up	miR-25-3p	Up	METTL3 promotes the maturation of miR-25-3p	promotes the initiation and progress of PDAC	(100)
	ALKBH5	Eraser	Down	LncRNA KCNK15-AS1	Down	ALKBH5 promotes the expression of lncRNA KCNK15-AS1	inhibits the migration and invasion of pancreatic cancer cells	(101)
	IGF2BP2	Reader	Up	LncRNA DANCR	Up	IGF2BP2 improves the stability of lncRNA DANCR	promotes pancreatic cancer cell proliferation, stem cell-like properties, and tumorigenesis	(71)
	HNRNPC	Reader	Up	has-miR-183-3p	–	rs7495 in HNRNPC 3’UTR disrupts the binding site of has-miR-183-3p, thereby increasing the expression of HNRNPC	promotes the proliferation of PDAC cells	(102)
Carcinoma of esophagus	WTAP	Reader	Up	lncRNA EMS	Up	LncRNA EMS promotes WTAP expression	promotes cisplatin resistance of esophageal carcinoma cells.	(103)
	ALKBH5	Eraser	Up	lncRNA LINC00278	Down	ALKBH5 inhibits LINC00278 encoded micropeptide YY1BM	promotes the progress of ESCC	(104)
Gallbladder cancer	METTL3	Writer	–	miR-2b-3p	Up	METTL3 promotes the maturation of miR-92b-3p	promotes the progression of gallbladder cancer	(105)

### Colorectal Cancer

Colorectal cancer (CRC) is one of the most common cancers of the digestive system, with the third highest incidence (10.0%) in the world, behind breast cancer (11.7%) and lung cancer (11.4%), and the second highest mortality (9.4%) in the world, only after lung cancer (18%) (106). Although traditional surgery combined with radiotherapy and chemotherapy has greatly improved the poor prognosis of colorectal cancer in recent years, the 5-year survival rate of colorectal cancer is still only 63.5%, and postoperative recurrence and liver metastasis of colorectal cancer are the main reasons for poor prognosis of patients with colorectal cancer (107, 108). Therefore, the exploration of the pathogenesis of colorectal cancer has a very important strategic significance. Through a comprehensive analysis of lncRNAs m^6^A modification in CRC, it was found that the methylation level of lncRNAs in CRC tissues was significantly higher than that in adjacent normal tissues (109); METTL14 promotes the m^6^A modification of the carcinogen lncRNA XIST, which makes XIST degrade in a YTHDF2-mediated manner, thereby inhibiting the proliferation and invasion of CRC cells (76); Similarly, METTL14 also acts on the tumor suppressor microRNA miR-375 and promotes the processing and maturation of miR-375. Finally, METTL14 inhibits the growth of CRC cells through the miR-375/YAP1 pathway and inhibits the migration and invasion of CRC cells through the miR-375/SP1 pathway (77); Epithelial-mesenchymal transition (EMT) is the first and most important step in cancer cell metastasis (110). METTL3-mediated m^6^A modification up-regulates the expression of lncRNA RP11. RP11 can trigger the migration, invasion, and EMT of colorectal cancer cells by promoting the post-translational up-regulation of the EMT transcription factor (EMT-TF) Zeb1 (70); The RNA-binding protein RALY (also known as hnRNPCL2) enhances the m^6^A modification of a subset of miRNAs (miR-483, miR-676, and miR-877) through METTL3, which promotes the post-transcriptional processing of a subset of miRNAs. These miRNAs systematically down-regulate the expression of metabolism-related genes (ATP5I, ATP5G1, ATP5G3, and CYC1), thereby reprogramming mitochondrial metabolism in colorectal cancer cells. Knockout of RALY gene can inhibit the growth and progression of colorectal tumors (73); METTL3 also up-regulates the expression of microRNA miR-1246 by promoting the maturation of miR-1246. miR-1246 negatively regulates the expression of the tumor suppressor gene SPRED2, thereby inactivating the Raf/MEK/ERK pathway and promoting the migration and invasion of CRC cells (69); LINC01605 is significantly overexpressed in CRC, and it could bind to METTL3 to promote m^6^A modification of SPTBN2 mRNA by METTL3, thus enhancing the translation of SPTBN2 mRNA. Overexpression of SPTBN2 leads to proliferation, migration, and invasion of CRC cells (74). In colorectal cancer, METTL3 induces m^6^A methylation of lncRNA LBX2-AS1 mRNA, thereby improving its mRNA stability and ultimately promoting its expression. LncRNA LBX2-AS1 is associated with proliferation, migration, invasion, and 5-FU resistance of colorectal cancer (75). In colon cancer, ALKBH5 can up-regulate the expression of lncRNA NEAT1 through demethylation modification, thereby promoting tumor progression. Moreover, ALKBH5 or NEAT1 gene knockout can partially inhibit the malignant behavior of colon cancer (78); CircNSUN2 is often upregulated in patients with liver metastasis (LM) from colorectal cancer and is exported from nucleus to cytoplasm in an m^6^A-dependent manner through binding to YTHDC1. CircNSUN2 in cytoplasm interacts with IGF2BP2 to form circNSUN2/IGF2BP2/HMGA2 RNA-protein ternary complex, thus enhancing the stability of HMGA2 (high mobility group AT-hook 2) mRNA. This ultimately leads to increased expression of HMGA2, which promotes liver metastasis from colorectal cancer (68); In CRC, lncRNA GAS5 directly binds to YAP and promotes YAP phosphorylation and ubiquitin-mediated degradation. This reduces YAP-mediated transcription of YTHDF3, thereby inhibiting CRC cell proliferation and invasion. On the contrary, YTHDF3 reversibly and selectively binds to the m^6^A modified GAS5 to trigger the decay of GAS5, thus promoting the progress of CRC and forming a negative feedback loop (79); In colorectal cancer, lncRNA LINRIS (Long Intergene Non-Coding RNA For IGF2BP2 Stability) is highly expressed. LINRIS blocks the degradation of Reader IGF2BP2 through the ubiquitination-autophagy pathway, thereby maintaining the stability of MYC mRNA (MYC mRNA is a typical target of IGF2BP2 and one of the core regulators of glycolysis) and promotes MYC-mediated Glycolysis and proliferation of colorectal cancer cells. This confirms the potential of LINRIS-IGF2BP2-MYC axis for colorectal cancer targeted therapy (80); LncRNA LINC00460 interacts with IGF2BP2 and DHX9 to form the LINC00460/DHX9/IGF2BP2 complex. This complex may increase the stability of HMGA1 mRNA by recognizing the m^6^A modification site of HMGA1 (high-mobility group at-hook 1), thereby enhancing the expression of HMGA1, and ultimately promoting the proliferation, migration, and invasion process of colorectal cancer cells (81).

### Gastric Cancer

The incidence of gastric cancer (GC) ranks fifth in the world, and it is also the fourth leading cause of cancer-related deaths in the world (106). The 5-year survival rate of patients with gastric cancer metastasis is less than 5% (111). Studies have found that in gastric cancer, the expression of METTL3 is elevated. METTL3 modifies the oncogene SEC62 mRNA by m^6^A, which promotes the stabilizing effect of IGF2BP1 on SEC62 mRNA and increases the expression of SEC62. Interestingly, miR-4429 inhibited the expression of METTL3, resulting in down-regulation of SEC62 expression, thereby preventing the progression of gastric cancer (84); LncRNA LINC00470 is significantly up-regulated in gastric cancer tissues and cell lines. LINC00470 enhances the modification of tumor suppressor PTEN mRNA by binding with METTL3. The up-regulation of m^6^A modification level of PTEN mRNA promotes the degradation of PTEN mRNA and decreases its expression, and finally promotes the proliferation, migration, and invasion of gastric cancer cells (82); In gastric cancer, the autophagy degradation of lncRNA ARHGAP5-AS1 is impaired, leading to its upregulation in chemotherapy resistant cancer cells. LncRNA ARHGAP5 -AS1 promotes the transcription of ARHGAP5 by interacting with the ARHGAP5 promoter and enhances the stability of ARHGAP5 mRNA in the cytoplasm by recruiting METTL3 to conduct m^6^A modification on ARHGAP5 mRNA. Finally, the expression of ARHGAP5 is up-regulated, which promotes the chemotherapy resistance of gastric cancer (83); LncRNA BLACAT2 is significantly upregulated in GC. LncRNA BLACAT2 can sponge miR-193b-5p, thereby blocking the inhibitory effect of miR-193b-5p on METTL3 and promoting the progression of GC (85). KIAA1429 up-regulated the expression of lncRNA LINC00958 in GC cells in an m^6^A dependent manner. LINC00958 promotes aerobic glycolysis of GC cells by enhancing the stability of GLUT1 mRNA (86); ALKBH5 promotes the high expression of lncRNA NEAT1 in gastric cancer cells and tissues through demethylation. NEAT1 can act as a scaffold to bind to EZH2 (a subunit of the multicomb-inhibiting complex), promote the expression of downstream genes of EZH2, and eventually lead to the invasion and metastasis of gastric cancer (87); MicroRNA miR-660, a tumor suppressor, is significantly reduced in gastric cancer tissues and cell lines. MiR-660 inhibits the expression of oncogene E2F3 (E2F transcription factor 3) by directly binding to E2F3 3’-UTR, and ultimately inhibits the proliferation of gastric cancer cells, in which m^6^A modification is a necessary condition for the interaction between miR-660 and E2F3 (112); In poorly differentiated gastric adenocarcinoma (PDGA), most of the differentiated expressed circRNAs (DECs) are modified by m^6^A, and the variation trend of m^6^A modification is basically consistent with the expression level of circRNAs. This suggests that the m^6^A modification of DECs may play a potential role in the progression of gastric cancer (113);

### Liver Cancer

Liver cancer is the third leading cause of death from tumors worldwide, causing more than 830,000 deaths each year (106). The high recurrence rate and high metastasis rate of liver cancer lead to poor prognosis, with a 5-year survival rate of less than 20%. Its symptoms are hidden, 80% of liver cancer patients are often found in the middle and late stage (114, 115). Hepatitis B virus (HBV) infection is an important cause of liver cancer. However, the pathogenesis of liver cancer is not fully understood, and the study of the molecular mechanism of liver cancer will be helpful to the development of new targeted drugs. Primary liver cancer includes hepatocellular carcinoma (HCC), intrahepatic cholangiocarcinoma (ICC), and HCC-ICC mixed type. The most common one is HCC, which accounts for about 90%. In HCC, METTL3 regulates the m^6^A modification level of lncRNA LINC00958, thereby increasing the stability of LINC00958 and upregulating its expression. LINC00958 promotes the proliferation, migration, invasion, and adipogenesis of HCC cells through miR-3619-5p/HDGF axis (88); In hepatoblastoma (HB), the expression of the tumor suppressor microRNA miR-186 is decreased. MiR-186 targets and negatively regulates METTL3 expression and inhibits the activation of Wnt/β-catenin signaling pathway, thereby inhibiting the proliferation and invasion of HB cells (72); In HBV-associated HCC, HBx protein encoded by HBV x gene upregulates METTL3 expression. This increases the m^6^A modification level of circ-ARL3. The combination of YTHDC1 and circ-ARL3 modified by m^6^A is conducive to reverse splicing and formation of circ-ARL3. Circ-ARL3 sponges miR-1305 and antagonizes the inhibition of miR-1305 on a group of target oncogenes, thereby promoting the progression of HBV^+^ HCC (90); LncRNA ILF3-AS1 expression is increased in HCC tissues. ILF3-AS1 increases the methylation level of ILF3 by recruiting METTL3, thereby inhibiting degradation of ILF3 and ultimately promoting the proliferation, migration, and invasion of HCC cells (91). In HCC, METTL3 enhanced the stability of lncRNA NIFK-AS1 mRNA by increasing the methylation modification level of NIFK−AS1, resulting in increased expression of NIFK-AS1. NIFK-AS1 can promote the growth and invasion of HCC cells and the resistance to sorafenib (92). LncRNA MEG3 is underexpressed in HCC tissues and cells. MEG3 can regulate the expression of BTG2 by sponging miR-544b, thus exerting its anticancer effect. Further studies found that METTL3 could inhibit MEG3 expression (93). It was found that the expression of KIAA1429, a key component of the m^6^A methyltransferase complex, is significantly up-regulated in HCC tissues. KIAA1429 regulates the expression of the tumor suppressor circDLC1, which is negatively correlated with the expression of circDLC1 in HCC tissues. CircDLC1 can bind to the RNA-binding protein Hur and reduce the interaction between Hur and MMP1 mRNA, thereby inhibiting the expression of MMP1 and ultimately inhibiting the progression of HCC (95); LncRNA GATA3-AS, transcribed from the antisense chain of GATA3 gene, specifically promotes the m^6^A modification of the tumor suppressor GATA3 precursor mRNA (pre-mRNA) by methyltransferase KIAA1429, which reduces the stability of GATA3 pre-mRNA. The decreased expression of GATA3 promoted the malignant phenotype of HCC cells (94); Additionally, miR-139-5p inhibits the EMT process of HCC by negatively regulating the expression of WTAP (96); Recent studies have shown that HCC is often associated with chronic inflammation. Lipopolysaccharide (LPS) stimulation promotes YTHDF1-mediated G-protein alpha-subunit (GNAS) translation in HCC cells by increasing the m^6^A modification of GNAS mRNA. The high expression of GNAS promotes the activation of STAT3 in LPS-induced HCC cells by inhibiting the interaction between the lncRNA TPTEP1 and STAT3, and ultimately led to the growth and invasion of LPS-induced HCC cells (97); The expression of circRNA circ_KIAA1429 is up-regulated in HCC. It can maintain the expression of ZEB1 through m^6^A-YTHDF3-ZEB1 mechanism, thereby promoting the migration, invasion, and occurrence of EMT in HCC cells (116); In sorafenib resistant HCC cells, the increased level of m^6^A modification of circRNA-SORE improves the stability of its RNA, thereby upregulating the level of circRNA-SORE. CircRNA-SORE acts as a miRNA sponge to isolate miR-103a-2-5p and miR-660-3p, and thus competitively activates the Wnt/β-catenin pathway, ultimately inducing sorafenib resistance in HCC (89);

### Pancreatic Cancer

Pancreatic cancer (PC) mainly originates from pancreatic ductal epithelial cells and follicular cells. The 5-year relative survival rate of pancreatic cancer is only 9%, which is one of the worst prognostic malignancies (117). A study in European countries predicts that pancreatic cancer will surpass breast cancer by 2025 and become the third leading cause of cancer death (106). In pancreatic cancer, m^6^A modification is highly enriched in LINC00857, which enhances its RNA stability and leads to up-regulation of its expression. LINC00857 acts as a ceRNA sponge to bind and inhibit miR-150-5p, resulting in enhanced expression of E2F3 (the target of miR-150-5p), thereby promoting PC cell proliferation and inhibiting apoptosis (98, 99); Cigarette smoke can induce the upregulation of METTL3 expression and the maturation of miR-25-3p by m^6^A modification. MiR-25-3p targets to inhibit the expression of tumor suppressor PHLPP2, thereby activating AKT-p70S6K oncogene signal and promoting the start and development of pancreatic ductal adenocarcinoma (PDAC) (100); ALKBH5 is down-regulated in pancreatic cancer tissues. It can demethylate lncRNA KCNK15-AS1, thereby promoting the expression of KCNK15-AS1. KCNK15-AS1 and ALKBH5 can inhibit EMT, thereby inhibiting the migration and invasion of pancreatic cancer cells (101); In pancreatic cancer, IGF2BP2 as a reader recognizes m^6^A-modified lncRNA DANCR, thereby improving the stability of DANCR, and ultimately promoting tumorigenesis, cell proliferation and stem cell-like properties of pancreatic cancer (71); The study found that rs7495 (SNP) in the 3’UTR of HNRNPC may destroy its binding site with has-miR-183-3p, thereby increasing the expression of HNRNPC and promoting the proliferation of PDAC cells (102);

### Carcinoma of Esophagus

Esophageal carcinoma is mainly divided into squamous cell carcinoma and adenocarcinoma, the most common clinical symptoms of progressive dysphagia. Esophageal cancer is the fourth leading cause of cancer death in China (118). Hypoxia leads to increased expression of lncRNA EMS in esophageal carcinoma. Overexpressed lncRNA EMS targets miR-758-3p, thereby increasing the expression of WTAP and ultimately promoting cisplatin resistance in esophageal cancer cells (103); Y-linked lncRNA LINC00278 encodes a micropeptide named YY1BM, which inhibits the expression of eEF2K by blocking the interaction between YY1(Yin Yang 1) and androgen receptor (AR). This led to the apoptosis of the cells of the Esophageal squamous cell carcinoma (ESCC). Smoking increases the expression of ALKBH5 and decreases the m^6^A modification level of LINC00278, thus inhibiting the translation of YY1BM and promoting the development of ESCC (104).

### Gallbladder Cancer

Gallbladder cancer (GBC) is the most common malignant tumor of the biliary tract. Although it is relatively rare, its median survival rate is only about 6 months (119). Deoxycholic acid (DCA) is down-regulated in GBC. DCA inhibits the maturation of miR-92b-3p by promoting the disintegration of METTL3 in the METTL3-METTL14-WTAP complex, thereby reducing the m^6^A modification level of pri-miR-92b in GBC cells. The reduction of miR-2B-3p leads to increased expression of the new miR-92B-3p target ——PTEN (phosphatase and tensin homolog) mRNA, thereby inhibiting the oncogenic PI3K/AKT signaling pathway in gallbladder carcinoma. This suggests that DCA acts as a tumor suppressor in GBC, and DCA therapy may provide a new therapeutic strategy for GBC (105).

## Conclusions and Perspectives

At present, there are more and more studies on RNA m^6^A modification, but the main focus is on the methylation of mRNAs, and less attention is paid to the methylation modification of ncRNAs. NcRNAs mainly include miRNAs, circRNAs, and lncRNAs, which can participate in protein expression through a variety of pathways. The interaction between m^6^A modification and ncRNA is related to the occurrence and progression of a variety of cancers. Therefore, the study of the interaction mechanism between m^6^A and ncRNA is conducive to the further development of related drugs for the treatment of tumors. METTL3 has been proved to be highly expressed in a variety of tumors and play a role in tumor progression, which provides ideas for the clinical application of METTL3 inhibitors. At present, METTL3 inhibitors have been identified as a promising anticancer therapy strategy in AML (acute myelogenous leukemia) (120, 121)

In this paper, the mechanism of the interaction between m^6^A and ncRNAs in gastrointestinal tract cancers was discussed in detail (**Table 2**), suggesting that: (1) m^6^A modification can affect the metabolism of ncRNAs, and similarly, ncRNAs can also affect the process of m^6^A modification, thereby regulating the occurrence and progression of tumors. For example, in colorectal cancer, METTL14 inhibits the proliferation and invasion of CRC cells by promoting the m^6^A modification of lncRNA XIST (76); lncRNA GAS5 inhibits the proliferation and invasion of CRC cells by reducing the transcription of YTHDF3 (79). (2) An m^6^A-related enzyme can influence tumor progression by regulating the metabolism of different ncRNAs. For example, METTL14 inhibits the proliferation and invasion of CRC cells by promoting the degradation of lncRNA XIST and the processing and maturation of microRNA miR-375 (76, 77); (3) The same molecule can play the opposite roles in different tumors. For example, METTL3 promotes the progression of CRC but inhibits the progression of gastric cancer (69, 70, 84). These findings reveal that the occurrence of tumors is co-regulated by multiple molecules and multiple pathways, and suggest that targeted therapies targeting multiple molecules and multiple pathways can be carried out simultaneously in the treatment of tumors.

Of course, in addition to the common miRNAs, circRNAs, and lncRNAs, ncRNAs also include snRNAs, snoRNAs, piRNAs, etc (122). The effect of the interaction between m^6^A and these ncRNAs on tumor development is still worthy of further study.

## Author Contributions

YF provided direction and guidance throughout the preparation of this manuscript. LY wrote and edited the manuscript. RH, LH, J-BH, S-YX, and ZD collected and prepared the related papers. X-YW and C-FM reviewed and made significant revisions to the manuscript. All authors contributed to the article and approved the submitted version.

## Funding

This study was supported by Jiangsu Innovative Team Leading Talent Fund (CXTDC2016006, QNRC2016446), Jiangsu 333 Talent Fund (BRA2020016),Jiangsu Provincial Key Research and Development Special Fund (BE2015666), Jiangsu Six High Peak Talent Fund (WSW-205, WSW236), Zhenjiang Key Research and Development Fund (SH2021038), Suqian Science and Technology Support Project Fund (K201907).

## Conflict of Interest

The authors declare that the research was conducted in the absence of any commercial or financial relationships that could be construed as a potential conflict of interest.

## Publisher’s Note

All claims expressed in this article are solely those of the authors and do not necessarily represent those of their affiliated organizations, or those of the publisher, the editors and the reviewers. Any product that may be evaluated in this article, or claim that may be made by its manufacturer, is not guaranteed or endorsed by the publisher.

## Glossary

ALKBH5: ALKB homolog 5
AR: androgen receptor
CRC: Colorectal cancer
CircRNAs: circular RNAs
DECs: differentiated expressed circRNAs
DCA: Deoxycholic acid
eIF3: eukaryotic translation initiation factor
EMT: Epithelial-mesenchymal transition
E2F3: E2F transcription factor 3
EMT-TF: EMT transcription factor
ESCC: Esophageal squamous cell carcinoma
FTO: fat mass and obesity associated protein
GC: Gastric cancer
GNAS: G-protein alpha-subunit
HBV: hepatitis B virus
HCC: hepatocellular carcinoma
HMGA1: high-mobility group at-hook 1
HMGA2: high mobility group AT-hook 2
HB: hepatoblastoma
HNRNP: heterogeneous nuclear ribonucleoprotein
ICC: intrahepatic cholangiocarcinoma
IGF2BP1/2/3: insulin-like growth factor 2 mRNA-binding protein 1/2/3
LINRIS: Long Intergene Non-Coding RNA For IGF2BP2 Stability
LM: liver metastasis
lncRNAs: long noncoding RNAs
LPS: Lipopolysaccharide
m^1^A: N^1^-methyladenosine
m^6^A: N^6^-methyladenosine
m^5^C: 5-methylcytosine
MA: Meclofenamic acid
miRNAs: micro RNAs
METTL3: methyltransferase-like protein 3
METTL14: methyltransferase-like protein 14
ncRNAs: non-coding RNAs
PTEN: phosphatase and tensin homolog
PDGA: poorly differentiated gastric adenocarcinoma
pre-mRNA: precursor mRNA
PC: Pancreatic cancer
PDAC: pancreatic ductal adenocarcinoma
SAM: S-adenosylmethionine
VIRMA: Vir like m6A methyltransferase associated
WTAP: Wilms tumor suppressor-1-associated protein
YY1: Yin Yang 1
3’-UTRs: 3’untranslated regions
